# How Do Intermolecular Interactions Evolve at the Nematic to Twist–Bent Phase Transition?

**DOI:** 10.3390/ijms231911018

**Published:** 2022-09-20

**Authors:** Katarzyna Merkel, Barbara Loska, Yuki Arakawa, Georg H. Mehl, Jakub Karcz, Antoni Kocot

**Affiliations:** 1 Institute of Materials Engineering, Faculty of Science and Technology, University of Silesia, ul. 75 Pułku Piechoty, 41-500 Chorzów, Poland; 2 Department of Applied Chemistry and Life Science, Graduate School of Engineering, Toyohashi University of Technology, Toyohashi 441-8580, Japan; 3 Department of Chemistry, University of Hull, Hull HU6 7RX, UK; 4 Faculty of Advanced Technologies and Chemistry, Military University of Technology, 00-908 Warszawa, Poland

**Keywords:** FTIR spectroscopy, DFT simulations, intermolecular interactions, liquid crystal dimers, twist–bend phase, self-assembling

## Abstract

Polarized beam infrared (IR) spectroscopy provides valuable information on changes in the orientation of samples in nematic phases, especially on the role of intermolecular interactions in forming the periodically modulated twist–bent phase. Infrared absorbance measurements and quantum chemistry calculations based on the density functional theory (DFT) were performed to investigate the structure and how the molecules interact in the nematic (N) and twist–bend (N_TB_) phases of thioether dimers. The nematic twist–bend phase observed significant changes in the mean IR absorbance. On cooling, the transition from the N phase to the N_TB_ phase was found to be accompanied by a marked decrease in absorbance for longitudinal dipoles. Then, with further cooling, the absorbance of the transverse dipoles increased, indicating that transverse dipoles became correlated in parallel. To investigate the influence of the closest neighbors, DFT calculations were performed. As a result of the optimization of the molecular cores system, we observed changes in the square of the transition dipoles, which well corresponds to absorbance changes observed in the IR spectra. Interactions of molecules dominated by pairing were observed, as well as the axial shift of the core to each other.

## 1. Introduction

One of the main goals of soft matter theory is to understand the structure–property relationship of material systems so that the specific properties of the materials can be obtained through molecular design. Self-organization is the process by which molecules spontaneously arrange themselves into stable and ordered structures due to noncovalent interactions. This process can occur on all scales, ranging from the nanoscopic, involving atoms or molecules, to cosmic-sized objects. These spontaneous organizations can deliver information on mounting components, i.e., their shape, charge, polarizability, magnetic dipole, mass, etc., as these properties determine their interactions [1]. Molecular self-assembly studies provide essential information about the influence of intermolecular interactions on the structure of the investigated molecular system. Therefore, such studies are crucial when anisotropic molecular systems can lead to direction-dependent physical properties. One of the fascinating examples of molecular self-organization is the emergence of chirality. The formation of chiral superstructures and phases is an area of great interest from theoretical and practical perspectives [2]. In the biological context, helical structures are found in DNA and proteins; twisted beta sheets form helical columns [3], similar to silk and its synthetic analogs reported in [4,5]. Materials science examples include cholesteric liquid crystalline structures [6] and chiral smectic phases [7], which are formed by bent-core molecules. The most famous example of the last decade of creating chiral structures using nonchiral molecules is the (spatially modulated) twist–bend nematic phase (N_TB_), which has a pitch length of several nanometers [8,9,10,11,12].

The experimental study of the structure of the N_TB_ phase has mainly been carried out with nonresonant (SAXS, WAXS) [13,14,15] and resonant X-ray scattering [10,11,16,17,18,19], freeze–fracture transmission electron and atomic force microscopy (FFTEM, AFM) [8,9,20,21,22], polarized Raman [23], infrared [24,25,26], and nuclear magnetic resonance spectroscopy (NMR) [27,28,29,30,31], as well as with optical methods under applied electric fields [32,33,34,35,36,37,38] and magnetic fields [39]. The formation of the N_TB_ structure has been modeled using rigid elements [15,40,41]. Some models [15,41] assume the nanophase segregation of the assembling elements: flexible central alkyl linker, terminal chains, and the mesogenic cores; molecular end groups with the flexible central spacers provide orientational freedom. Finally, the X-ray evidence of the half-molecular length periodicity along the N_TB_ helix led to a model based on the self-assembly of half-molecule-long blocks as the basic element of the N_TB_ phase [10,18,42]. The N_TB_ materials that have been most investigated are the cyanobiphenyl (CB)-based liquid crystal dimers (CBCnCB; *n* = 7, 9, 11) [8,9,10,11,13,16,21,23,27,28,29,30,31,32,33,36,37,38,39], where cyanobiphenyl units prefer an antiparallel arrangement due to dipole–dipole interactions, [43,44,45,46]. Up-to-date research has confirmed that molecular curvature is important for phase formation, but its stability increases as the bending angle decreases [47,48,49,50,51,52]. There have also been important reports suggesting that such factors also influence the N–N_TB_ phase transition as intramolecular torsion [17,31,53], conformational changes [54,55,56,57,58], bend angle fluctuations [59], the effect of free volume [54,60,61], as well as intermolecular interactions [25,62,63,64,65,66].

It has long been known that hydrogen bonds and the π–π intermolecular interactions play a crucial role in supramolecular biological phenomena such as base-pairing in double-stranded DNA, protein binding, cell–cell recognition, and viral infection [67,68]. A similar phenomenon is observed in the field of soft matter, where unconventional intermolecular interactions lead to the emergence of new supramolecular liquid crystal structures [69,70], such as the twist–bend phase (N_TB_) [71] or the polar–twisted phase (N_PT_) [72], as well as the newly discovered ferroelectric nematic phase (N_F_) [73,74].

This work is devoted to observations of the intermolecular interactions and their impact on the structural changes that occur during the transition from the nematic phase to the twist–bend phase. For this purpose, infrared spectroscopy was an excellent tool for analyzing orientational order in a molecular system. In the case of conventional nematics, it is usually assumed that the transition dipole moments that correspond to a specific vibration are temperature-independent. As absorbance is related to the square of the transition dipole moment, the average absorbance is expected to be density-dependent. Density, in turn, is typically temperature-dependent. In the N_TB_ phase, however, the average absorbance of several molecular vibrations exhibits a specific behavior due to the molecular system’s self-assembly. Experimental data indicate that short-range interactions grow significantly, and thus the intercorrelations between the transition dipoles of the interacting functional groups become more important. Moreover, the longitudinal dipoles’ behavior differs from those of the transversal dipoles.

In the paper, we report combined FTIR and DFT studies of intermolecular interaction for two groups of dimers: a symmetric system with the thioether-linking groups (C-S-C) termed CBSCnSCB (*n* = 5, 7) and an asymmetric system with the ether- and thioether-linking groups, termed CBSCnOCB (*n* = 5, 7). A symmetric dimer with ether-linking groups (C-O-C) with the acronym CBOCnOCB (*n* = 7) is chosen for reference.

## 2. Results

### 2.1. Temperature Dependencies of the Absorbance

By measuring the absorbance in polarized light, the temperature dependencies of the absorbance components in the temperature range of the N and the N_TB_ phases could be directly analyzed [24,25,26]. The experiment will distinguish the different behavior of the absorbance components for the bands with longitudinal and transverse dipoles in the molecular frame. In an orientationally ordered material, the absorbance components are dependent on the angle between the alignment axis and the polarization direction of the incident beam. At a microscopic level, the infrared absorption depends on the angle between the molecular transition dipole moment μ_i_ of the particular absorption band and the polarization of the IR beam. The average IR absorbance A_0_ = (A_X_ + A_Y_ + A_Z_)/3 of the specific vibrational modes is determined by how the electric dipole moment of the system changes with the atomic oscillations. To the lowest order, the required quantities are proportional to the derivatives of the dipole moment with respect to the system’s vibrational normal modes, i, evaluated at the equilibrium geometry. The IR absorbance of the i_th_ vibrational mode is given by [75]:

(1) $$A_{i}=\overset{\nu 2}{\underset{\nu 1}{\int }}A\left(\nu \right)\mathrm{dv}=\frac{n\pi }{3c}{\left[\frac{{d\mu }_{i}}{{\mathrm{dQ}}_{i}}\right]}^{2}$$

where n is the number of molecules per unit volume (molecular density) and μ_i_ is the molecule transition dipole moment for the normal coordinate, Q_i_, corresponding to the ith mode.

The temperature dependence of the average absorbance, A_0_, and the corresponding transition dipoles was analyzed for all dimers in the N and the N_TB_ phases. In the range of the N phase, the average absorbance increased with the molecular density, which confirmed that the transition dipole seemed to be constant in this temperature range. At temperatures below the N_TB_ transition, the behavior of symmetric and asymmetric dimers was different and, in some cases, was determined by cell windows [26].

**Figure 1:**
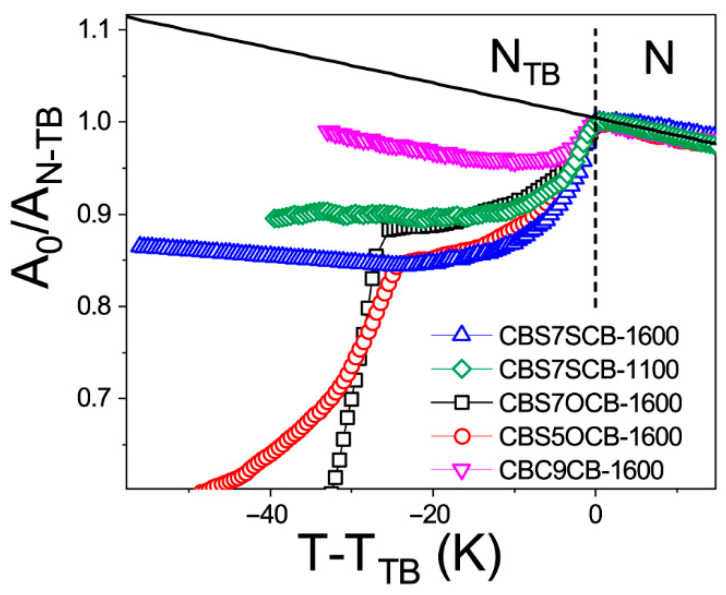
Normalized IR absorbance vs. temperature behavior of the dimers for the longitudinal dipole (μ): benzene ring vibration at 1600 cm^−1^ (νCC) and a deformation vibration of the C-H group in the benzene ring plane at 1100 cm^−1^ (βCH ip CB + ν_as_C_Ar_S). ☐–CBSC7OCB, ◯–CBSC5OCB, **△**–CBSC7SCB (1600 cm^−1^), and **▽**–CBC9CB; **◇**–CBSC7SCB (1100 cm^−1^); solid black line—nematic phase trend line. A_0_—integral average absorbance (cm^−1^), A_N-TB_—integral absorbance in the transition from nematic to twist–bend phase (cm^−1^).

A detailed analysis of the infrared spectra compared to those simulated for different conformers, along with the exact assignments of vibrations, is presented in a separate paper [76]. Several vibrational bands that belonged to the longitudinal and transverse transition dipole that showed significant dichroism of the band were selected to be analyzed. For the longitudinal transition dipole moment, the phenyl stretching band (νCC) at 1600 cm^−1^ and the C-H deformation vibration in the benzene plane at 1100 cm^−1^ (βCH) were selected. The band at 1100 cm^−1^ was a vibration that involved a sulfide group, so it may be a good indicator of the dimer’s conformational change [76]. Figure 1 shows the temperature dependencies of the average absorbances, A_0_, for the bands corresponding to the longitudinal transition dipole. We introduced the nematic phase trend line as a reference: the solid black line in Figure 1. There was a clear transition from the conventional N phase to the N_TB_ for all of the molecules, except for CBOC7OCB, which behaves like a classical calamitic molecule. This is attributed to its large opening angle and which is responsible for not forming the N_TB_ phase. The absorbances involving the biphenyl group (1600 cm^−1^ and 1100 cm^−1^ in Figure 1), and also the band at 2220 cm^−1^ (that involved a cyan group), show a significant decrease just after entering the N_TB_ phase (see Table 1). This indicates the structure rearrangement in the N_TB_ phase, resulting in the antiparallel orientation of the neighboring cyanobiphenyl groups. Both dipole–dipole interaction and London dispersion forces are likely involved in this process that stabilizes the N_TB_ phase. It seems the nanosegregation process proceeds along the *Z*-axis of the system. A similar conclusion has already been presented for cyanobiphenyl-based LCs, showing an overlap of the termini of the molecules [44,45,46,61]. Following the transition to the N_TB_, a significant decrease in the average absorbance was measured, suggesting the presence of dipole correlations in this phase and the presence of other molecular interactions related to the change in the system’s geometry.

We noticed that, for the CBC9CB dimer, there was a significantly smaller decrease in the mean absorbance for the band at 1600 cm^−1^ than for the other dimers. This indicates the importance of the sulfide group in this phenomenon.

**Figure 2:**
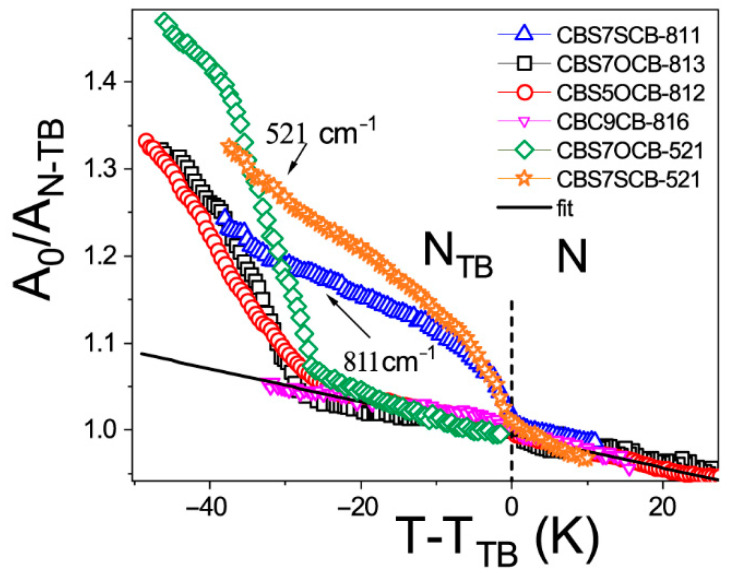
Normalized IR absorbance vs. temperature behavior of the dimers for the transversal dipole (μ_⊥_): 811 (813/816) cm^−1^ (γCH op CB for the rigid core with a sulfur linkage C−S−C): ☐–CBSC7OCB, ◯–CBSC5OCB, **△**–CBSC7SCB, and **▽**–CBC9CB; 520 cm^−1^ (γCC op CB + δCS): **◇**–CBSC7OCB, **✯**–CBSC7SCB; solid black line—nematic phase trend line. A_0_—integral average absorbance (cm^−1^), A_N-TB_—integral absorbance in the transition from nematic to twist–bend phase (cm^−1^).

Three bands were identified as a probe for transverse interaction: these are out of the benzene plane of the C-C vibration, which also involved a thioether bridge (γCC op CB + δCS; op—out of benzene plane) at 520 cm^−1^; the second one was assigned to the out of plane deformation vibrations of the C-H groups at 811 cm^−1^ (γCH op CB), the complex band at 821 cm^−1^ for CBSCnOCB dimers (γCH op CB + ν_s_ C_Ar_O; s–symmetric), and the in-plane deformation of the C-H groups at 1395 cm^−1^ (βCH ip CB with a sulfur linkage C-S-C). Figure 2 shows the temperature dependencies of the average absorbance, A_0_, for the transversal transition dipole moment. We introduced the nematic phase trend line as a reference: the solid black line in Figure 2.

For a symmetric dimer (CBSC7SCB), the average absorbance of the transversal dipole is affected by the transition to the N_TB_ phase. However, in contrast to the longitudinal ones, they show a significant increase in their trend for all transversal dipoles. For the asymmetric dimers (CBSC7OCB), however, two steps of the temperature changes in the N_TB_ phase could clearly be distinguished (for transversal dipoles of sulfur linkages 811 and 521 cm^−1^): the first extending almost 30 K below the N–N_TB_ transition temperature and the second below this range. In the first region, the absorbance continued to increase as the molecular density increased to nearly 30 K below the N–N_TB_ transition temperature. Then, the absorbances of the sulfur linkages (811 and 521 cm^−1^) started to grow significantly. This was conveyed by the further absorbance decrease of the longitudinal dipole (Figure 1). Such behavior indicates the emergence of another interaction which causes so-called bond orientation and an escape from the uniaxial arrangement [26]. Please note the absorbance for CBC9CB is not affected by the N–N_TB_ transition. All the above can prove the leading role of the sulfur group in the emergence of bond orientations. They are involved in the π–π stacking interactions and in hydrogen bonds between neighbors. It should be pointed out that the vibration that involved the sulfur group (~811 cm^−1^) showed an absorbance increase, while the other with the oxygen group (~820 cm^−1^) did not.

The latter result can be analyzed considering the rotation barrier between the cyanobiphenyl group’s bond and the alkyl linker. This barrier is much higher for the oxygen group than for the sulfur one [12,76], so it can be assumed that the oxygen bridge remains in planar conformation. However, the sulfur bridge has conformational freedom and can escape from the phenyl plane [76]. It is reasonable to conclude that the origin of the absorbance increase is due to transversal order rearrangement and emergence phase biaxiality.

In light of the experimental results, we attempted to investigate possible intermolecular interactions resulting in bond orientation. To determine the molecular rearrangement on entering the N_TB_ phase, we performed density functional calculations (DFT) for the system, including the nearest lateral neighborhood. The calculated polarized IR spectra for such a system allow determining the transition dipole moments of particular vibration to control a specific type of interaction that might influence them.

### 2.2. Intermolecular Nonspecific Binding Energy in the System

Regarding the vibrations of individual molecular groups, we find the contributions of the couplings between vibrations for the rigid mesogenic cores within one molecule are negligible, in other words, hair-pin conformations are very rare [24,25,26]. On the other hand, the interactions of the mesogenic groups belonging to adjacent dimers turn out to be significant [25,26]. Therefore, to confirm the experimental observations and determine the molecular arrangement in the N_TB_ phase, DFT simulations were performed for the system, including the nearest lateral neighborhood. With the focus on following neighbor interactions and considering that DFT simulations for such large molecules are very time-consuming, calculations were directed at the CBSC7 and the structurally related CBOC7 monomers, judged to show the relevant features for adjacent interactions. First, the optimization of the single molecules was performed. The potentially stable conformers were determined based on calculating the energy barriers for the internal rotation of the cyanobiphenyl group (CB) and the rotation around the dihedral angle between the CB and the linker. All the technical details were described and discussed in a previous paper [76].

**Figure 3:**
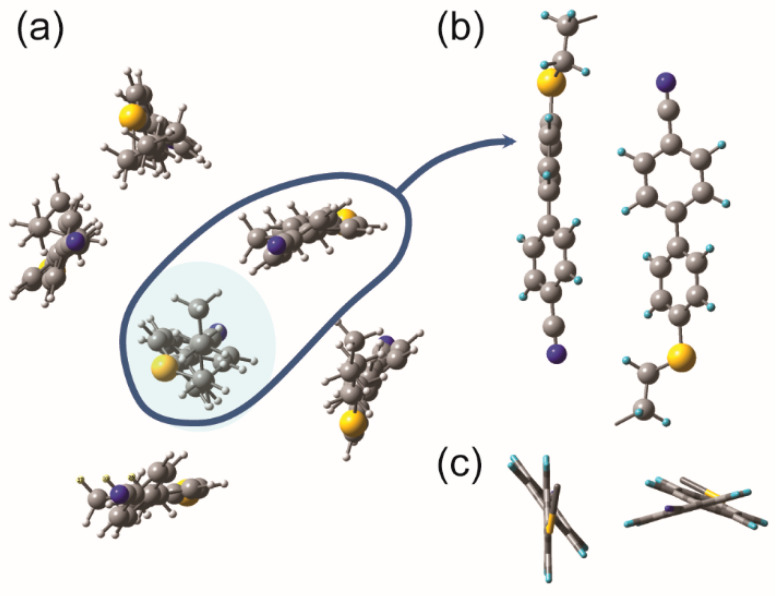
View of the CBSC7 monomer components. (**a**) Top view (X-Y plane) of the system with the initial arrangement of six molecules into a sublayer prepared for DFT calculations. IR spectra were calculated for a molecule (highlighted in blue) that is surrounded by other molecules. (**b**) The close-up view of a CBSC7 molecule pair after optimization using the B3LYP/6-311 G (d, p) method. (**c**) Top view (X-Y plane) of a pair of molecules (stick representation of the bonds).

In the next step, the six monomer molecules consisting of the optimized molecule and the most energetically stable conformer [76] were arranged in a so-called sublayer (Figure 3a). The arrangement of the molecules in such a sublayer was created based on the detailed experimental data from resonant X-ray scattering measurements (TReXS) collected for these molecules [12].

We prepared two systems: one that contains only CBSC7 molecules and a hybrid system consisting of three CBSC7 and three CBOC7 molecules. Figure 3a shows, for example, a top view of the initial arrangement of the CBSC7 molecule selected for calculation. Molecules were arranged parallel to each other, the distance between them was set at approximately 5Å, and their cyan groups were arranged alternately. The system was optimized using the B3LYP/6-311 G (d, p) method. In the next stage for the optimized system, the atoms and bonds of the molecules located at the system’s periphery were frozen. The frequencies and intensities of the vibrations were calculated for a central molecule that was surrounded by other molecules (Figure 3a, the selected molecule highlighted in blue). This approach allowed the calculation of the FTIR spectrum and, more precisely, the square of the dipole transition moment for a single molecule, taking into account the influence of the nearest surroundings. The binding energy was determined using the so-called supramolecular approach using the superposition error basis set (BSSE) correction utilizing the counterpoise method (CP). It was estimated at 30 and 37 kJ/mol (for the thioether and hybrid systems, respectively).

(2) $$\Delta E^{CP}=E^{AB}-E^{A\left(AB\right)}-E^{B\left(AB\right)}$$

where $E^{AB}$ is the energy of the complex, $E^{A\left(AB\right)}$—the energy of fragment A, which was calculated in the dimer base and, $E^{B\left(AB\right)}$—the energy of fragment B, which was calculated in the base of the dimer. Figure 3b,c show a group of the molecules into pairs.

### 2.3. Hypothetical Arrangement of the CBSC7SCB Molecules—DFT Modeling

In order to determine the molecular arrangement in the N_TB_ phase for the system, including the nearest lateral neighborhood, the geometry of the system was optimized based on the arrangement of the six interacting molecules. These are the monomers with a sulfur atom CBSC7, Figure 3a, and the hybrid system containing the CBSC7 and the CBOC7 molecules. After optimization, it was observed that the distance between the rigid mesogens decreased (ranging from 4.1 Å to 4.7 Å), which corresponds very well with the average lateral intermolecular distance determined from X-ray scattering measurements (~4.5 Å) for such molecules [12]. The differences are mainly due to ignoring/neglecting the thermal aspect in this DFT simulation. A group of the molecules were arranged into pairs and a shift of the molecular axes relative to each other was detected (see Figure 3b). As observed in numerous physical experiments, a nanosegregation into pseudolayers–biphenyls “stacking” next to each other, creating an aromatic pseudolayer, and the spacer tending to be parallel to form an aliphatic layer was observed as well. The molecules with a sulfide bridge are coaligned so that the phenyl planes of cyanobiphenyl remain in the so-called “T-shape” conformation (Figure 3c) relative to their neighbors. In the case of oxygen bridged molecules, a flatter “parallel-displaced” arrangement was observed. The segregation of the aromatic parts (partial overlapping) and an attraction of the CN group to the alkyl chains were observed. Molecular interdigitating also occurred [44,46].

**Figure 4:**
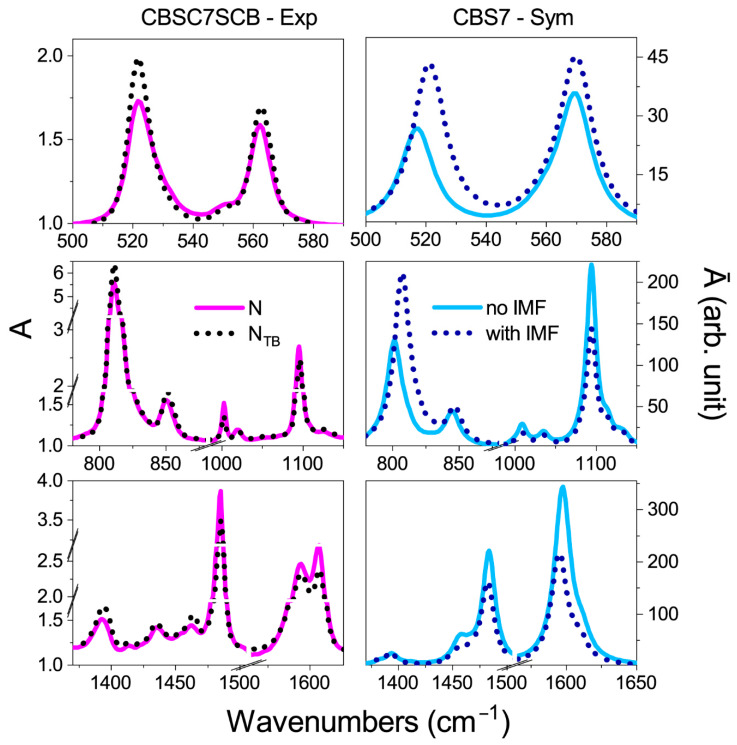
Comparison of the theoretical and experimental spectra for the symmetric dimer. Left Panel: Experimental spectra of the CBS7SCB dimer, solid magenta line—the nematic phase, short black dotted line—the twist–bend nematic phase (spectrum is represented for 5 μm cell). Right Panel: Simulations for the CBS7 monomer: solid blue line—spectra for an isolated molecule (no IMF), short navy dotted line—a system of six interacting molecules (with IMF).

**Figure 5:**
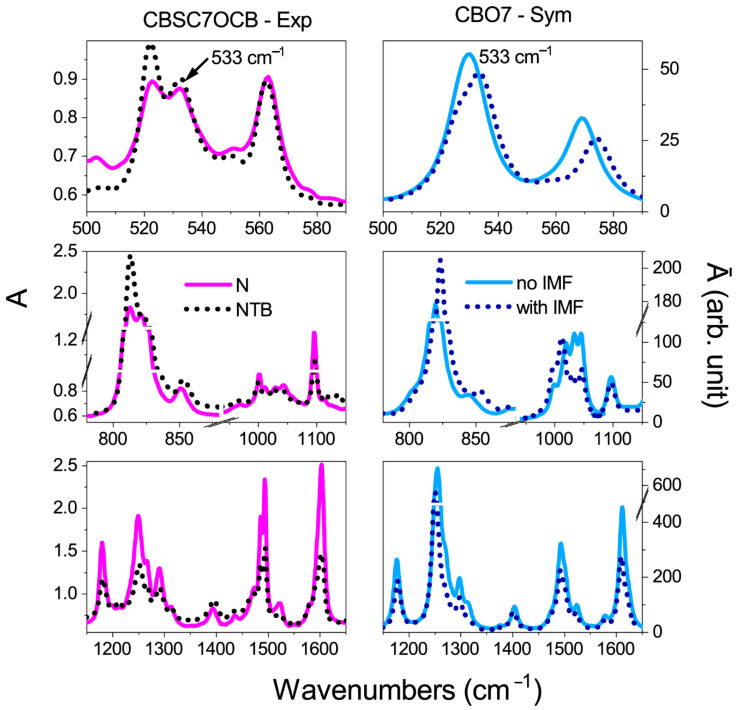
Comparison of the theoretical and experimental spectra for the asymmetric dimer. Left Panel: Experimental spectra of the CBS7OCB dimer, solid magenta line—the nematic phase, short black dotted line—the twist–bend nematic phase (spectrum is represented for 5 μm cell). Right Panel: Simulations for the CBO7 monomer: solid blue line—spectra for an isolated molecule (no IMF), short navy dotted line—a system of six interacting molecules (with IMF).

In the next stage, the outer molecules were frozen, while the vibrational frequencies and transition dipole moments were calculated for the molecule that remained in the center (see Figure 3a). The theoretical IR spectrum for a surrounded molecule should reflect the situation in which the intermolecular forces (IMF) are considered. The IR spectrum of a molecule where the intermolecular forces were considered was compared with the spectrum of the isolated molecule and the experimental one in the N and N_TB_ phases (Figure 4 and Figure 5).

For the longitudinal dipoles (bands at wavenumbers: 1000, 1100, 1485, 1600, and 2220 cm^−1^), a decrease in the intensity of the bands for the system with the IMF when compared to a single monomer was detected. This result is consistent with the correlation along the Z-direction, visible in the IR experiments. An increase in the intensity for the transverse bands was also observed, i.e., for the bands at 520 and 811 cm^−1^. This result implies a correlation of the transverse dipoles in the direction perpendicular to the nematic order (so call bond ordering).

For bands with transversal dipoles (520, 811 cm^−1^), an increase in the intensity of the bands by approximately 50% relative to an isolated molecule was detected. For the vibrations in the longitudinal dipoles (1100, 1485, 1600 cm^−1^), the decrease was approximately 40%. The slightest change in intensity was obtained for the CN vibrations at 2220 cm^−1^ (about 10%). These values align with the trend of absorbances observed in experimental spectra at the transition from the N to the N_TB_ phase. Table 1 summarizes the absorbance changes in the transition from the N to the N_TB_ phase (related to the square of the corresponding transition dipole moments) with a calculated ratio $\mu_{IMF}^{2}/\mu_{noIMF}^{2}$ in the presence of weak intermolecular interactions and local orientational order.

We note that the interactions between several molecules do not yet fully explain the behavior of a group of molecules. Thermodynamic and probabilistic considerations related to entropy and enthalpy changes must be considered to determine this behavior fully.

Hence, our current calculations are to be viewed as the first step toward more advanced simulations using periodic density functional theory modeling, which we plan to perform in the future.

## 3. Discussion

**Figure 6:**
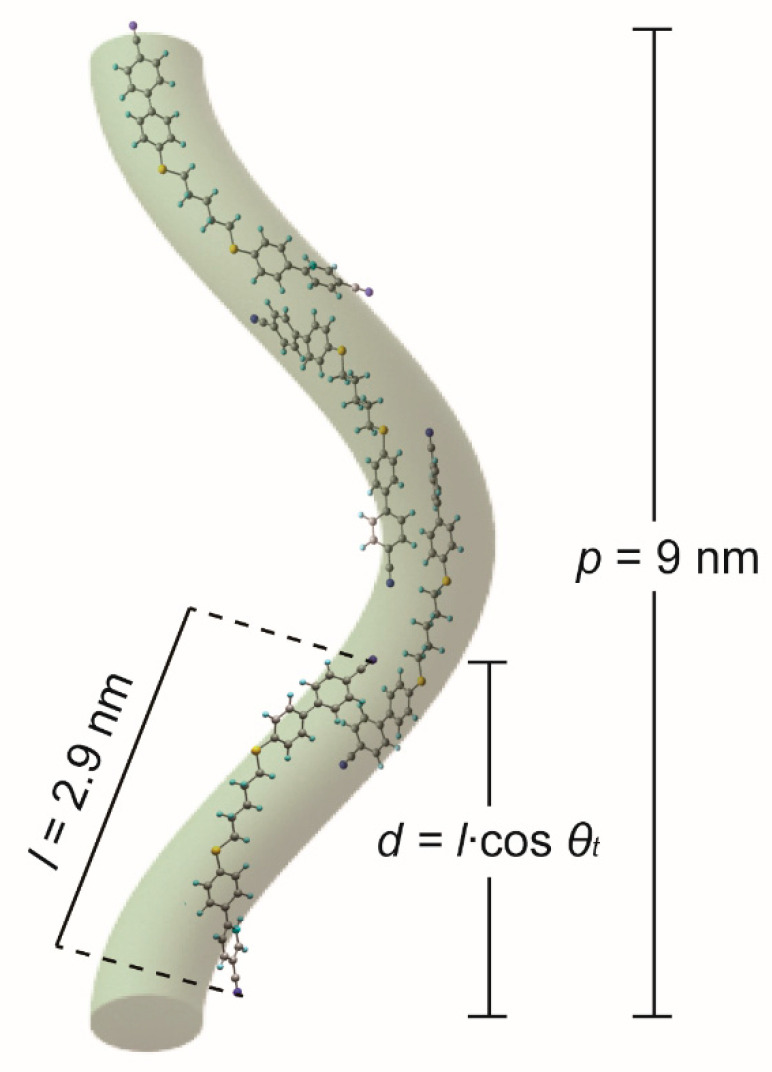
Proposed hypothetical arrangement of the thioether dimers in the twist–bend nematic phase. The helix pitch was computed as *p* = 2π/*q*, where *q* is the wave vector.

Based on the analysis of the molecular structure, a model of the geometrical arrangement of the molecules in the N_TB_ phase was developed (Figure 6). In this model, the molecules follow the shape of the helix formed by the director, with overlapping the CB groups formed. This is due to increased bond and dipole correlations, as shown through the optimization of the geometry of the monomer system. We found that for the investigated dimers, the hydrogen bonds between the CN group and the hydrogen atoms of the spacer, the hydrogen bonds between the sulfur atom and the hydrogen atom of the benzene ring, and the interactions of the π–π orbitals of the benzene rings all play an essential role. The presence of intermolecular interactions in the N_TB_ phase is clearly visible. The model and the experimental results for intermolecular interactions are consistent with the reports of other measurements obtained using the TReXS (Tender Resonant X-ray Scattering) method [11,12]. Table 2 shows the summary of the determined molecular parameters.

## 4. Materials and Methods

### 4.1. Materials

**Figure 7:**
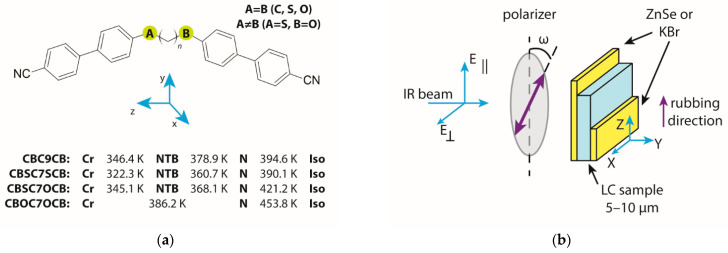
(**a**) Transition temperature and molecular structure of the cyanobiphenyl dimers with a molecular frame of reference: z—long axis (bowstring), x—axis normal to the bent plane, y—bow arrow axis. (**b**) Schematic of the polarized infrared transmission technique at a normal incidence of light. The planar cell’s laboratory frame (X, Y, Z). In the nematic phase, Z was the axis along and X was perpendicular to the optical axis (the optical axis coincided with the rubbing direction). In the N_TB_ phase, Z coincided with the helix axis.

Liquid crystal dimers were investigated based on the cyanobiphenyl (CB) mesogenic groups. We represent symmetrical dimers by the general abbreviation CBAC7BCB with A = B, where A and B = C, S or O (see Figure 7a). In the asymmetric dimers with the acronym CBSCnOCB (*n* = 5,7), the mesogens were linked to an alkyl spacer on one side by a thioether bridge and the other by an ether one. The primary CBC9CB compound was synthesized as described in Refs. [77,78,79,80]. All details on the synthesis of thioether dimers and the preliminary result of differential scanning calorimetry (DSC) and X-ray scattering studies are summarized in papers [81,82]. Figure 7a shows the molecular structure of the CB dimers.

### 4.2. Infrared Spectroscopy

The planar-aligned cells were prepared between two optically polished zinc selenide (ZnSe) windows. The windows were spin-coated with a SE-130 commercial polymer aligning agent (Nissan Chemical Industries, Ltd., Pasadena, TX, USA) in order to obtain a homogeneous alignment. The cells were assembled with a parallel positioning of the rubbing direction, and Mylar foil was used as a spacer to provide 2 μm and 5 μm separation. The thickness of the cells was determined by the measurements on the interference fringes using a spectrometer interfaced with a PC (Avaspec-2048). The samples were capillary filled by heating an empty cell in the N phase, five degrees below the transition to the isotropic phase. The quality of the alignment was tested using polarizing microscopy. The textures of the samples were monitored using a polarizing microscope (Olympus BX56). The spectra were acquired using a Fourier infrared spectrometer (Agilent Cary 670) with a resolution of 1 cm^−1,^ and these spectra are averaged over 32 scans. The experiment was conducted in the transmission mode with a polarized IR beam. An IR-KRS5 grid polarizer was used to polarize the IR beam. The IR spectra were measured as a function of the polarizer rotation angle in the wavenumber range 500–4000 cm^−1^. To keep the absorbance in the linear regime, we combined the spectrum of a 5 μm sample with a spectrum of 2 μm near strong bands of the spectra (810 cm^−1^).

The measurements were performed using slow cooling and heating at a rate of 0.5 K/min. For the samples with a thioether bridge (CBSC7SCB, CBSC7OCB), an additional measurement was taken with a faster cooling rate (4 K/min) in a temperature range from a few degrees above the N–N_TB_ transition until the solid phase (glass or crystal) was obtained. A faster cooling rate near the N–N_TB_ transition temperature significantly increased the width of the N_TB_ phase range for sulfide-bridged samples, so the later results were selected in the data analysis. The temperature of the samples was stabilized using a PID temperature controller with an accuracy of 2 mK.

Figure 7b shows the configuration of the infrared measurements using the polarized transmission technique. These measurements enabled the orientation of the transition dipole moment of the bands to be determined with respect to the long molecular axis and the temperature dependencies of the absorbance of the samples. To determine all components of absorbance (A_X_, A_Y_, and A_Z_), it was necessary to measure two cells with different orientations: planar (homogeneous) and homeotropic. Unfortunately, in the case of the tested materials, i.e., for the cyanobiphenyl dimers, it was extremely difficult to obtain a good homeotropic alignment. Therefore, to calculate the mean absorbance, assuming that the material was uniaxial and A_X_ = A_Y_, the mean absorbance was determined as A_0_ = (2A_X_ + A_Z_)/3. The absorbance components were determined as being the area bound by the contour of the given band using Bio-Rad Win-IR Pro version 2.96e. In the case of complex bands that contained more vibrations, they were separated using the Origin Pro 2021 software using the Pearson VII fit.

### 4.3. Density Functional Theory Calculations

In this work, the molecules’ electronic structure calculations were performed using the Gaussian09 software package (version E.01) [83]. The molecular structures, binding energy, harmonic vibrational force constants, absolute IR intensities, and components of the transition dipole moments were calculated using the density functional theory (DFT). The Becke’s three-parameter exchange functional combined with the Lee, Yang, and Parr correlation functional was applied (B3LYP) with the polarization basis set (6-311 (d, p)). [84,85].

Information about the components of the transition dipole moment for a specific vibration enables the parallel and perpendicular components of the spectral density to be calculated. The parallel component of the absorption coefficient was calculated as the square of the component of the transition dipole moment along the axis that coincided with the long axis of the dimer ${\left|\mu_{z}\right|}^{2}=\mu_{∥}^{2}$. To determine the perpendicular component of the spectral density, the sum of the squares of the transition dipole moments along the vertical directions was used ${\left|\mu_{x}\right|}^{2}+{\left|\mu_{y}\right|}^{2}=\mu_{⊥}^{2}$. The direction of the transition dipole moment was determined according to the molecular reference system (Figure 7a).

The theoretical vibrational frequencies were scaled by one coefficient equal to 0.98 to simplify the comparison with the experiment, and the Gaussian profile was used with a 7 cm^−1^ full width at half maximum (FWHM). The results were visualized using GaussView 6.

## 5. Conclusions

The study’s most striking finding was the evidence that the intermolecular forces evolved on the transition from the nematic (N) phase to the twist–bend (N_TB_) phase. Changes in the behavior of intermolecular interactions were observed by significant differences in the values of transition dipole moments for selected vibrational bands. The longitudinally induced dipoles of the CB group showed negative correlations due to the antiparallel mesogen arrangement, while the perpendicular dipoles were positively correlated; in other words, they increased. To explain this phenomenon of such self-organization, the DFT modeling was performed for the system, taking into account the nearest side neighborhood. Results that is most consistent with the experimental data is a system of several molecules that exhibits a clustering of the rigid CB cores and nonspecific weak intermolecular interactions. These interactions are mainly associated with the π−π orbitals interactions of the aromatic rings and the sulfur atoms. However, the formation of weak hydrogen bonds H⋅⋅⋅S/O and H⋅⋅⋅N may also be present. The nonspecific intermolecular interactions resulted in a significant bond ordering in the N_TB_ phase.

Based on the DFT simulation for the groups of interacting molecules, along with the experimental data from absorbance measurements, as well as X-ray resonance scattering, a model for the packing of the dimer molecules in the twist–bend phase was developed with overlapping of the rigid cyanobiphenyl cores stabilizing the N_TB_ phase and thus affecting the helical pitch.

## Figures and Tables

**Table 1:** The absorbance changes on transition from the N to the N_TB_ with calculated ratio $\mu_{\mathrm{IMF}}^{2}/\mu_{\mathrm{noIMF}}^{2}$ in the presence of weak intermolecular interactions and local orientational order.

Vibration Frequency (cm^−1^)	A_0_/A_N-TB_	$\mu_{IMF}^{2}/\mu_{noIMF}^{2}$ *	A_0_/A_N-TB_	A_0_/A_N-TB_	$\mu_{IMF}^{2}/\mu_{noIMF}^{2}$ *	A_0_/A_N-TB_
	CBSC7SCB	CBSC7SCB	CBC5OCB	CBC7OCB	CBC7OCB	CBC9CB
520	1.25	1.60	1.42	1.48	1.54	--
810	1.22	1.64	1.32	1.33	1.41	1.05
1100	0.94	0.65	0.94	0.84	0.84	--
1250	--	--	0.89	0.71	0.80	--
1485	0.89	0.73	0.90	0.66	0.70	0.94
1600	0.86	0.63	0.78	0.60	0.61	0.93
2220	0.80	0.93	0.85	0.68	0.95	0.93

* DFT simulation—B3LYP/6-311 G (p, d).

**Table 2:** Summary of the determined molecular parameters: the length of the molecule (*l*), the tilt angle (*θ_t_*), the helix pitch (*p*), the effective length of the molecule (*d*), and the number of molecules per helix pitch (*p*/*d*).

CBACnBCB	*θ_t_* (°)	*p* (nm)	*l* (nm) *	*d = l*cos *θ_t_*	*p*/*d*
CBC9CB	25.6	8.15 [10]	2.88	2.6	3.1
CBSC7SCB	33	9.1 [11,12]	2.90	2.43	3.7
CBSC7OCB	15.6	11.5 [12]	2.90	2.79	4.1

*—lengths of the molecules were determined for the optimized molecules using the B3LYP/6-31 G (d, p) method (CBC9CB–U conf., CBSC7SCB–F conf., CBSC7OCB–F conf., see paper [76]).

## Data Availability

The data will be made publicly available when all research results are published. All data stored on the RUJ will have a DOI number and consist of the project name, person and project manager, and date of project duration. https://ruj.uj.edu.pl/xmlui/, accessed on 1 October 2021.
